# Risk-assessment and risk-taking behavior predict potassium- and amphetamine-induced dopamine response in the dorsal striatum of rats

**DOI:** 10.3389/fnbeh.2014.00236

**Published:** 2014-07-15

**Authors:** Sara Palm, Shima Momeni, Stina Lundberg, Ingrid Nylander, Erika Roman

**Affiliations:** Neuropharmacology, Addiction and Behaviour, Department of Pharmaceutical Biosciences, Uppsala UniversityUppsala, Sweden

**Keywords:** addiction, behavioral profiles, chronoamperometry, multivariate concentric square field™ (MCSF), Multivariate data analysis, neurochemistry, open field, partial least squares projections to latent structures (PLS)

## Abstract

Certain personality types and behavioral traits display high correlations to drug use and an increased level of dopamine in the reward system is a common denominator of all drugs of abuse. Dopamine response to drugs has been suggested to correlate with some of these personality types and to be a key factor influencing the predisposition to addiction. This study investigated if behavioral traits can be related to potassium- and amphetamine-induced dopamine response in the dorsal striatum, an area hypothesized to be involved in the shift from drug use to addiction. The open field and multivariate concentric square field™ tests were used to assess individual behavior in male Wistar rats. Chronoamperometric recordings were then made to study the potassium- and amphetamine-induced dopamine response *in vivo*. A classification based on risk-taking behavior in the open field was used for further comparisons. Risk-taking behavior was correlated between the behavioral tests and high risk takers displayed a more pronounced response to the dopamine uptake blocking effects of amphetamine. Behavioral parameters from both tests could also predict potassium- and amphetamine-induced dopamine responses showing a correlation between neurochemistry and behavior in risk-assessment and risk-taking parameters. In conclusion, the high risk-taking rats showed a more pronounced reduction of dopamine uptake in the dorsal striatum after amphetamine indicating that this area may contribute to the sensitivity of these animals to psychostimulants and proneness to addiction. Further, inherent dopamine activity was related to risk-assessment behavior, which may be of importance for decision-making and inhibitory control, key components in addiction.

## Introduction

Substance use disorders are heterogeneous with regard to etiology, liability for addiction and response to treatment (Agrawal and Lynskey, 2008; Ducci and Goldman, 2012). Several risk factors have been identified, including personality traits such as sensation seeking and impulsivity (Labouvie and Mcgee, 1986; Conway et al., 2002; Franques et al., 2003; Terracciano et al., 2008).

Sensation seeking is positively correlated to risk-taking behaviors such as exploration in response to novelty, impulsive decision-making and extravagance in approach to reward cues (Wills et al., 1994; Laviola et al., 1999; Zuckerman and Kuhlman, 2000; Franques et al., 2003). Increased novelty seeking/risk taking has been associated with predisposition to rewarding and addictive behaviors (Laviola et al., 1999; Belin and Deroche-Gamonet, 2012).

Locomotor response in a novel environment can be used to classify animals as either high responders (HR) or low responders (LR) and has been used in several studies investigating individual differences in drug-related behavior (Piazza et al., 1989; Dellu et al., 1996; Kabbaj, 2006; Blanchard et al., 2009). HR rats display enhanced sensitivity to behavioral sensitization and locomotor effects of psychostimulants and more readily acquire self-administration of these drugs than LR rats (Piazza et al., 1989; Hooks et al., 1991; Pierre and Vezina, 1997; Marinelli and White, 2000; Klebaur et al., 2001; Mantsch et al., 2001). Higher dopamine activity has also been found in HR rats compared to LR (Piazza et al., 1991; Antoniou et al., 2008). Comparative studies between characteristics of HR rats and sensation-seeking humans have noted several similarities, including altered dopaminergic activity (Blanchard et al., 2009). Further, a rapid decline in the stimulatory effect of novelty in rats is similar to human sensation-seekers' susceptibility to boredom (Dellu et al., 1996).

Although drugs of abuse have different mechanisms of actions, they all increase dopamine levels in the nucleus accumbens acutely after intake (Di Chiara and Imperato, 1988). The transition from initial drug use to compulsive use and addiction involves long-lasting changes in neural networks (Koob and Volkow, 2010) and is hypothesized to involve a shift from the acute reinforcing effects in the nucleus accumbens to compulsive intake and recruitment of the dorsal striatum (Everitt and Robbins, 2013). The dopaminergic activity in the dorsal striatum could therefore also influence vulnerability to drug addiction.

In the current study, we used a combination of animal experimental techniques previously not used in the investigations of individual differences in drug-induced response. Instead of dividing the animals solely on locomotor activity, a risk-taking component was added by basing the division of the animals on activity in the inner part of a novel open field (Löfgren et al., 2009; Momeni et al., 2014). The first aim of the study was to investigate how this division related to the more widely used HR/LR classification. The second aim of the study was to investigate if risk-taking behavior in the open field would correlate with risk-taking behavior in the multivariate concentric square field™ (MCSF) test (Meyerson et al., 2006). If so, this would further strengthen the use of the MCSF in future studies, with the advantage of a more diverse behavioral profile without repeated testing.

Similar to the HR rats, high risk-taking rats show increased operant responding to food reward (Davis et al., 2009) possibly connected to differences in dopaminergic activity. The third aim was therefore to investigate dopaminergic differences of the risk-taking classification. This was done using a chronoamperometric method recording potassium chloride evoked dopamine release *in vivo* in the dorsal striatum, alone or in combination with systemic amphetamine. Further, the fourth aim was to investigate if behavioral parameters outside of the risk-taking behavior would correlate with dopaminergic activity and give an indication of other behavioral traits that could be potential risk factors for drug addiction.

## Materials and methods

### Ethics statement

All animal experiments were approved by the Uppsala Animal Ethical Committee and followed the guidelines of the Swedish Legislation on Animal Experimentation (Animal Welfare Act SFS1998:56) and the European Communities Council Directive (86/609/EEC).

### Animals

Thirty outbred male Wistar rats (RccHan™:WI, Harlan Laboratories B.V., Horst, The Netherlands) arrived at the animal facility at 7 weeks of age. The animals were housed in groups of three in cages (59 × 38 × 20 cm) with pellet food (Type R36; Lantmännen, Kimstad, Sweden) and tap water *ad libitum*. The cages contained wood-chip bedding, a wooden house and paper sheets (40 × 60 cm; Cellstoff, Papyrus) and were changed twice a week by animal care personnel. The animal room was kept at constant temperature (22 ± 1°C) and humidity (50 ± 10%) on a reversed 12 h dark/light cycle with lights off at 06:00 A.M. The test rooms were kept at similar conditions as the animal room and all rooms had a masking background noise to minimize unexpected sounds that could disturb the animals.

### General procedure

The animals arrived in batches of six once a week and all the tests were performed on a running schedule so that all the animals were age matched in each test. Animals were allowed 2 weeks of acclimatization before testing began. The third week they were handled on three occasions, consisting of picking the animal out of its home cage to be gently stroked, weighed and adapted to a bucket used to transport the animals from the animal room to the testing room. During the fourth and fifth week the open field and MCSF tests, respectively, were performed. An overview of the general procedure can be found in Figure S1. All behavioral tests were performed during the dark period of the dark/light cycle and carried out by the same person. After each animal, the arenas were wiped with 10% alcohol solution and allowed to dry before the next animal was tested. On the sixth week chronoamperometric recordings were performed by another investigator and the animals were then euthanized by decapitation. A median split of the duration (%) in the inner part of the open field was used to classify all animals as either low (LRT) or high risk taking (HRT) and was done after all recordings of behavior and chronoamperometric measurements had been finished. The experimenter doing the chronoamperometric recordings was therefore blinded to the behavioral classification of the animal.

### Behavioral testing

#### The open field test

The open field arena was black and circular (90 cm in diameter) enclosed by walls (35 cm high) in black stain-less steel and a black stain-less steel wire-mesh floor (10 mm between bars). The level of illumination in the center was 100 lx (Momeni et al., 2014). The arena was divided into zones, i.e., the center (C; 30 cm in diameter) surrounded by an inner circle (IC; width 15 cm), which was surrounded by an outer circle (OC; width 15 cm). The test started by placing the rat in the outer circle facing the wall and each rat was given 20 min to explore the arena. The percentage of time spent in the inner circle and center (%D IC + C), i.e., the duration (%) in the inner part of the open field, was used for classification of risk-taking behavior, thus dividing rats by central activity vs. thigmotaxis. One animal was excluded from the classification due to technical problems, leaving a total of 29 animals.

#### The multivariate concentric square field™ test

The multivariate concentric square field™ (MCSF) test is an ethologically founded test and unlike many other common tests, the MCSF test is unprejudiced with regard to mental condition allowing for a more diverse behavioral profile. The MCSF test arena, the general testing procedure and the behavioral recording have been described in detail elsewhere (Meyerson et al., 2006; Roman and Colombo, 2009). The entire arena (100 × 100 cm) is divided into several qualitatively different zones, that encourage exploration of zones associated with risk assessment, risk taking, exploration and shelter seeking, and form the basis of the description and the variables of the animals' performance in this test (Figure S2). The light conditions (lx) in the arena were as follows: dark corner room <1; central circle approximately 20; corridors and hurdle <5–10; slope approximately 30; bridge 600–650. The test started with placing the rat in the center facing the wall between the center and bridge and the animal was then allowed to explore the arena for 20 min. An operational categorization of the various parameters with regard to function (i.e., general activity, exploratory activity, risk assessment, risk taking and shelter seeking) is used in the interpretation of results (Meyerson et al., 2013).

Three animals were excluded from the MCSF analysis, leaving 27 animals. One animal was the previously unclassified rat from the open field, and two were excluded due to incomplete recordings.

#### Behavioral recordings

All behavior tests were recorded by video cameras placed above the arenas and observed from an adjacent room. Rearing and grooming in the open field and MCSF, and stretch attend postures (SAPs, standing with hind legs in one of the corridors stretching out the body into the central field) in the MCSF was recorded by direct observation. After each test in the MCSF the photocell counts of head dips into the holes in the hurdle hole-board were noted as well as the number of urinations and defecations. Manual scoring was done using the software Score 3.3 (Copyright Soldis, Uppsala, Sweden) to obtain latency (L, s) to first visit, frequency (F) and duration (D, s) of visits to the different zones. Ethovision version 2.3 (Noldus Information Technology Inc., Wageningen, The Netherlands) was used for automatic tracking in order to obtain the mean velocity (cm/s) and total distance (cm) traveled.

### Chronoamperometric recordings of dopamine *in vivo*

#### Materials

Inactin®, Nafion® 5% solution, dopamine hydrochloride, L-ascorbic acid, potassium chloride, sodium chloride, sodium phosphate, calcium chloride and d-amphetamine sulfate from Sigma-Aldrich, LLC (St Louis, MO, USA). Kerr sticky from DAB LAB AB (Upplands Väsby, Sweden). Carbon fiber microelectrodes (SF1A; 30 μm outer diameter × 150 μm length) from Quanteon, LLC (Nicholasville, KY, USA), silver wire (200 μm, Teflon-insulated) from A-M Systems Inc. (Carlborg, WA, USA), glass capillaries (0.58 mm inner diameter) for the micropipettes from World Precision Instruments Ltd (Stevenage, UK).

#### Surgery

Surgery was performed immediately prior to the electrochemical recordings. Animals were anesthetized with Inactin® 125 mg/kg intraperitoneally and placed in a stereotaxic frame (Stoelting Co., Wood Dale, IL, USA). A water-circulating heating pad (Gaymar Industries, Inc., Orchard Park, New York) was used to maintain body temperature. Two holes in the skull were drilled, one for the microelectrode, and another remote from the recording site for the reference electrode.

#### Chronoamperometric recordings of dopamine release and uptake

High-speed chronoamperometric measurements (1 Hz sampling rate, 200 ms total) were performed using the FAST16-mkII recording system (Fast Analytical Sensing Technology, Quanteon, LLC, Nicholasville, KY, USA) according to a procedure described previously (Littrell et al., 2012). Carbon fiber microelectrodes (SF1A) were heated for 5 min at 200°C and then coated with three coats of Nafion® with 5 min heating at 200°C after each coating (Gerhardt and Hoffman, 2001). The electrodes were then calibrated *in vitro* in 0.05 M phosphate buffered saline to determine selectivity, limit of detection (LOD) and slope before use *in vivo* (Littrell et al., 2012). The microelectrodes showed linear responses to serial additions of dopamine (2–6 μM), with an average correlation coefficient (R2) of 0.998 ± 0.0003. The following average (± s.e.m.) values were obtained: selectivity for all electrodes used in this study was 18506 ± 3462 μM for dopamine over ascorbic acid, LOD was 0.028 ± 0.003 μM dopamine, slope was −1.04 ± 0.06 nA/μM dopamine, and the reduction/oxidation ratio measured during the reference peak responses of dopamine was 0.66 ± 0.03, which is indicative of the detection of predominantly dopamine (Gerhardt and Hoffman, 2001). The reference electrode for *in vivo* use was prepared by plating a silver wire (Lundblad et al., 2009).

#### *In vivo* experimental protocol

A micropipette (10–15 μm inner tip diameter), filled with KCl solution (120 mM KCl, 29 mM NaCl, 2.5 mM CaCl_2_ • 2H_2_O; pH 7.2–7.4), was affixed 150–200 μm from the microelectrode tip using sticky wax. The electrode was placed in the dorsal striatum, AP: +1.0 mm, L: +3.0 mm (Paxinos and Watson, 2007), initially dorsal (−3.0 mm) to the recording site, using a micromanipulator (Narishige International Ltd, London, UK), and allowed to baseline for 45–60 min before being lowered to −4.0 mm. The electrode was then allowed to stabilize before the effect of a single injection of KCl on dopamine release was determined (Lundblad et al., 2009; Miller et al., 2012). KCl was applied using pressure ejection (10–20 psi for 0.5–1.0 s) controlled by a PicoSpritzer® III (Parker Hannifin Corporation, Pine Brook, NJ, USA) adjusted to deliver 100 nl KCl, measured using a surgical microscope fitted with an eyepiece reticule (Friedemann and Gerhardt, 1992).

Three reference peaks similar in amplitude were produced, 10 min apart. Five min later, rats were given a subcutaneous injection of amphetamine (2 mg/kg) or saline (1 ml/kg). After 5 min, KCl-induced release was evoked every 10 min producing peaks at 5, 15, 25, 35, 45, 55, and 65 min after the systemic injection (Figure S3A).

#### Verification of electrode placement and exclusions

Electrodes were cut and left in place after the experiment and placement was verified by sectioning the frozen brains. Twenty-eight animals were recorded and two were excluded due to wrongful placement, three due to recording errors and three due to electrical disturbances. Correlation with behavior was investigated in 17 animals, i.e., animals with complete data from both behavioral tests and the chronoamperometry.

### Data analysis

Statistical analyses were performed using Statistica 10 (StatSoft Inc., Tulsa, OK, USA) and the significance level was set to *p* < 0.05. For the multivariate data analysis, SIMCA-P + 12.0.1 (Umetrics AB, Umeå, Sweden) was used and partial least squares projections to latent structures (PLS) were created according to a previously published method (Wold et al., 2001).

#### Behavioral testing

The parameters in the open field and MCSF tests were not normally distributed, as shown by the Shapiro-Wilk's test, and were therefore assessed using non-parametric statistics. For comparison between the low risk taking (LRT) and high risk taking (HRT) groups the Mann-Whitney *U*-test was used. Occurrences were tested using the Chi-squared test.

A trend analysis was also used to analyze the performance in the MCSF, see Meyerson et al. (2013) for further details. In the trend analysis, behavioral parameters for each individual within the population were ranked. The rank values were then summed into functional categories; general activity (total activity, frequency and duration/frequency in all corridors and frequency in center), exploratory activity (duration in all corridors, center and hurdle, rearing, and photocell counts), risk assessment (duration/frequency on slope and bridge entrance and stretch attend postures to center), risk taking (frequency, duration, and duration/frequency on the bridge and in the central circle) and shelter seeking (frequency, duration, and duration/frequency in the dark corner room). Differences between the groups were then tested with the Mann-Whitney *U*-test.

Multivariate data analysis was used to investigate the relationship between behavioral parameters from the open field and the MCSF tests. A principal component analysis (PCA) of all behavioral data was made and the loading plot from this guided further investigations of correlations. For example, variables that load close together in a PCA usually show high positive correlation with each other. Correlations were confirmed using Spearman rank order correlations.

#### Chronoamperometric recordings

Maximal amplitude and time for the peak to decline to 80% of its amplitude (T80) (Figure S3B) were calculated using the FAST Analysis software version 4.4 (Quanteon, LLC, Nicholasville, KY, USA). The percentage of the reference peaks was calculated for the peaks following systemic injection. The amphetamine group was further divided into LRT and HRT. A repeated measures ANOVA, comparing the saline and the two amphetamine groups over time, was used followed by Fisher's LSD *post-hoc* test. Relationship between amplitudes and T80 values and behavioral data was investigated using multivariate data analysis, followed by Spearman rank order correlations.

## Results

### Behavioral tests

#### Classification

A median split of the duration (%) in the inner part of the open field was used to classify all animals as either LRT or HRT. The median was 6.2% (range 2.3–16.3%). Representative traces from the open field can be found in Figure S4.

#### The open field test

Differences between the LRT and HRT rats were found in several parameters, (Table S1), including duration (%) in the inner part (*U* = 0.0; *p* < 0.001), used to classify the animals. The HRT animals had higher total activity (*U* = 17.0; *p* < 0.001), reared significantly more (*U* = 53.0; *p* = 0.02) and traveled both longer (*U* = 54.0; *p* = 0.03) and faster (*U* = 55.5; *p* = 0.03) in the arena and longer in the center (*U* = 22.0; *p* = 0.03) and inner circle (*U* = 31.0; *p* = 0.001) than the LRT rats.

**Figure 1:**
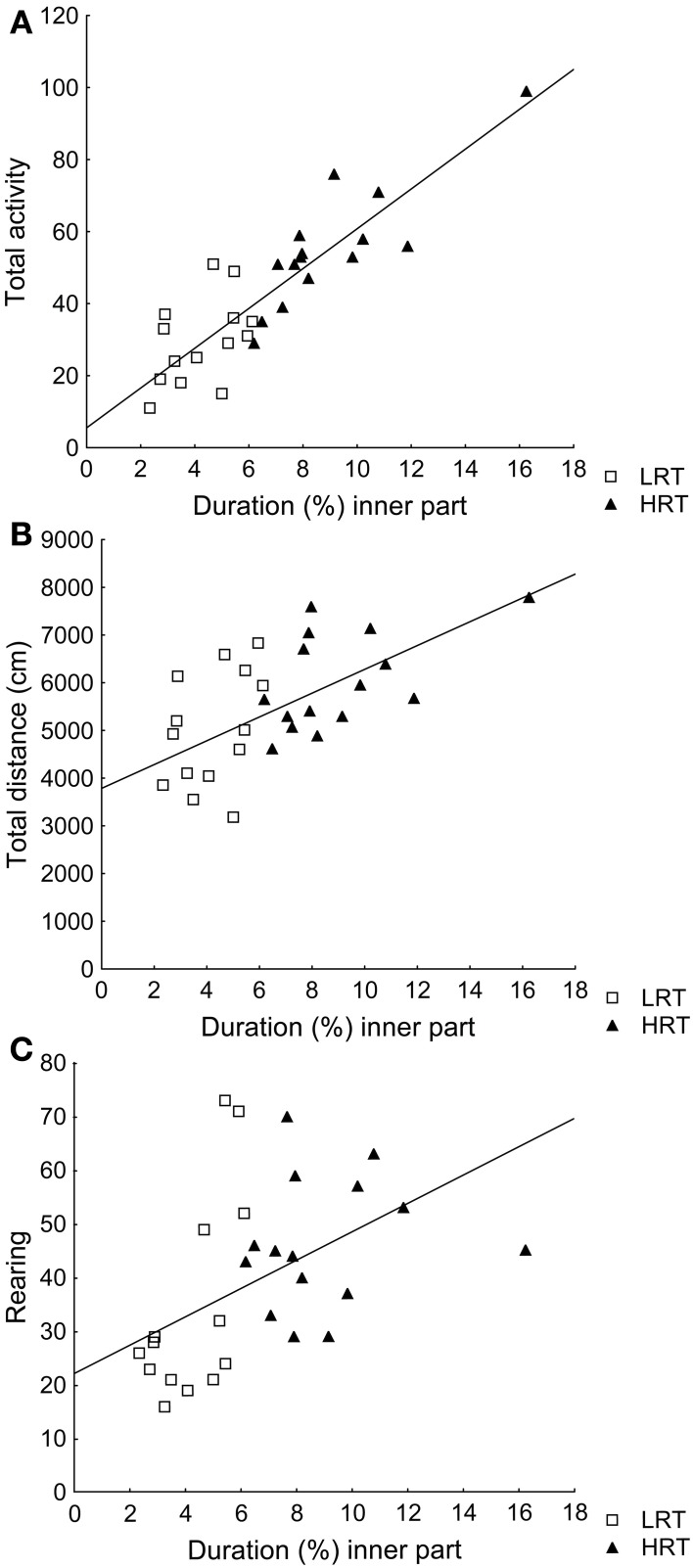
**Spearman rank order correlations between the parameter duration (%) in the inner part of the open field, used to classify the animals into the low (LRT, *N* = 14) or high risk taking (HRT, *N* = 15) groups, and (A) the total activity [ρ = 0.85; *p* < 0.001], (B) the total distance traveled [ρ = 0.59; *p* < 0.001], and (C) rearing [ρ = 0.60; *p* < 0.001] in the open field test**.

Correlations were found between the duration (%) in the inner part and total activity (ρ = 0.85; *p* < 0.001), total distance (ρ = 0.59; *p* < 0.001) and rearing (ρ = 0.60; *p* < 0.001), Figures 1A–C.

#### The multivariate concentric square field™ test

The HRT rats had higher frequency (*U* = 48.5; *p* = 0.04), duration (*U* = 47.0; *p* = 0.03) and percent duration (*U* = 47.0; *p* = 0.03) in the central circle, used for interpretation of risk-taking behavior, compared to LRT rats (Table S2).

**Figure 2:**
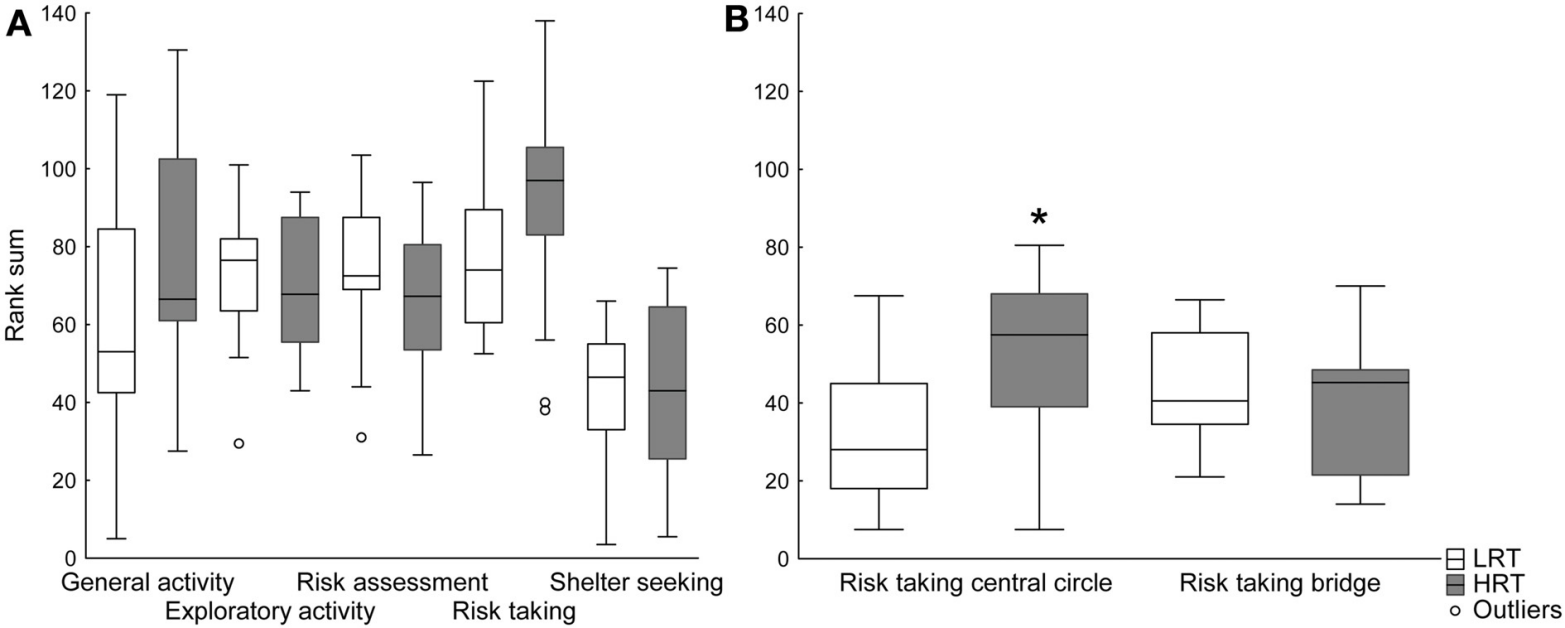
**Trend analysis of (A) all functional categories and (B) the risk taking category divided into central circle- and bridge-related parameters in the low (LRT, *N* = 13) and high risk taking (HRT, *N* = 14) groups in the multivariate concentric square field™ (MCSF) test**. Data are shown as boxplots with the median and 25–75 percentiles and non-outlier range. ^*^p < 0.05 compared to the LRT group. General activity, total activity, frequency and duration/frequency in all corridors and frequency in center; exploratory activity, duration in all corridors, center and hurdle, rearing and photocell counts; risk assessment, duration/frequency on slope and bridge entrance and stretch attend postures to center; risk taking, frequency, duration and duration/frequency on the bridge and in the central circle; shelter seeking, frequency, duration and duration/frequency in the dark corner room.

***Trend analysis***. The trend analysis did not reveal any differences between the groups, Figure 2A. However, when dividing the risk-taking category into central circle-related parameters (frequency, duration and duration per frequency in the central circle), and bridge-related parameters (frequency, duration and duration per frequency on the bridge), the risk taking in the central circle was significantly higher in the HRT compared to the LRT (*U* = 43.0; *p* = 0.02) rats, Figure 2B.

#### Correlations between the open field and MCSF parameters

The PLS did not produce any significant components, but guided further Spearman correlations confirming that duration (%) in the inner part of the open field correlated with the trend analysis category risk taking (ρ = 0.44; *p* = 0.021), and that the correlation was stronger when risk taking connected to the central circle of the MCSF was used (ρ = 0.49; *p* = 0.010) (Figure S5). Risk taking connected to the bridge did not correlate with duration (%) in the inner part of the open field (ρ = −0.002; *p* = 0.99). Central circle parameters such as frequency (ρ = 0.48; *p* = 0.011) and duration (ρ = 0.43; *p* = 0.028) also showed significant correlation with duration (%) in the inner part of the open field, data not shown.

### Dopamine recordings

#### Reference values

In a repeated measures ANOVA, with three groups (saline, amphetamine LRT and amphetamine HRT) and three time points (reference 1, 2, and 3), no statistically significant effects of group [*F*_(2, 17)_ = 0.2; *p* = 0.82] or time [*F*_(2, 34)_ = 0.1; *p* = 0.87] were found for the reference amplitudes (Table S3). For the three reference T80 values, no effect of group [*F*_(2, 17)_ = 0.4; *p* = 0.67] or time [*F*_(2, 34)_ = 2.4; *p* = 0.11] was found (Table S3).

#### Amphetamine response

***Amplitudes.*** In the response of the amplitudes to amphetamine or saline, a main effect of time [*F*_(6, 96)_ = 4.7; *p* < 0.001] was found (Table S4). No effect of group [*F*_(2, 16)_ = 0.8; *p* = 0.46] or any interaction was found [*F*_(12, 96)_ = 0.9; *p* = 0.57].

**Figure 3:**
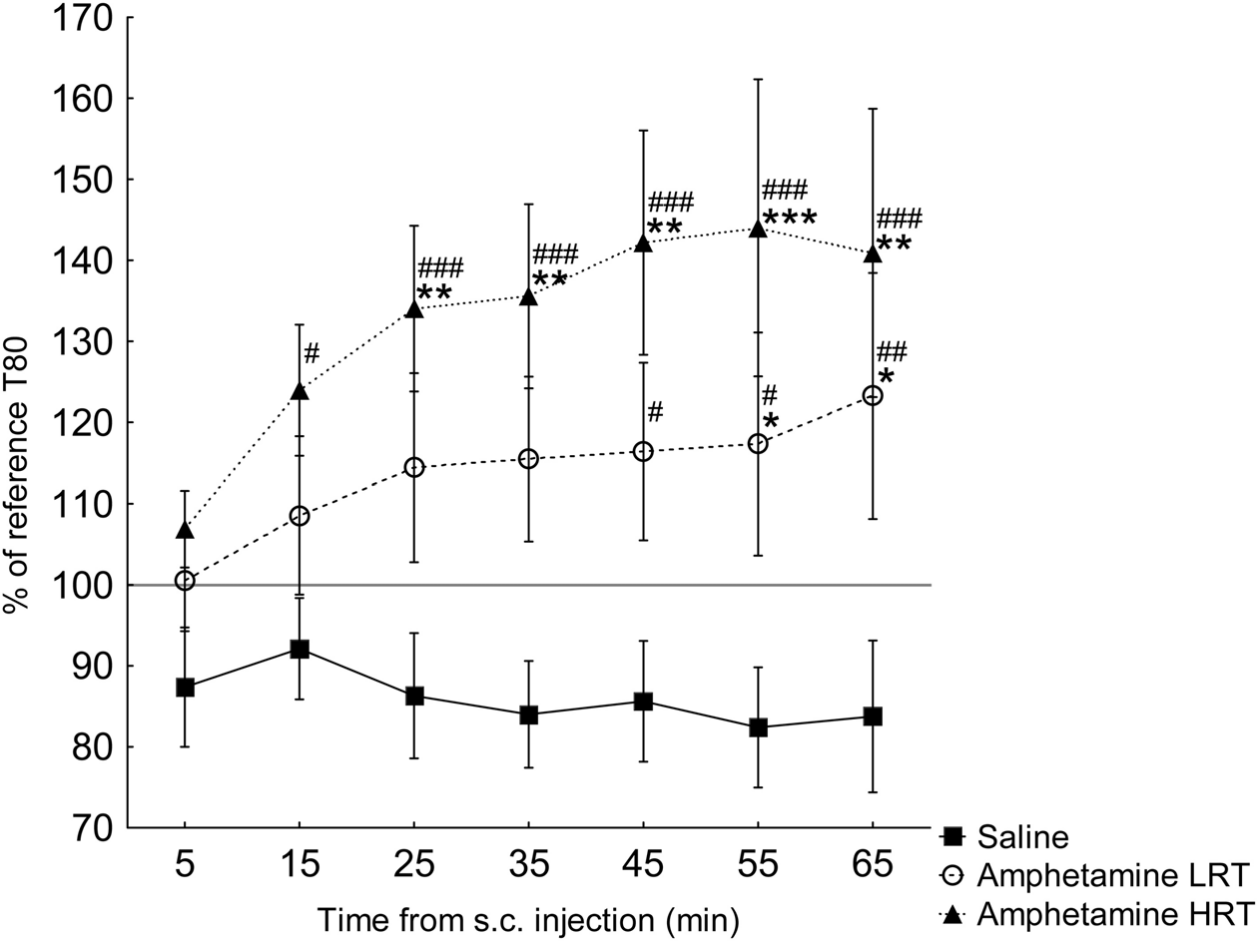
**The response in T80 (mean ± s.e.m.) to a subcutaneous (s.c.) injection of amphetamine or saline (*N* = 7) shown as the percent of the reference T80**. The amphetamine animals were further divided into low (LRT, *N* = 6) and high risk taking (HRT, *N* = 7) groups. ^*^p < 0.05, ^**^p < 0.01, ^***^p < 0.001 compared to the saline controls; ^#^*p* < 0.05, ^##^*p* < 0.01, ^###^*p* < 0.001 compared to the 5-min time point.

***T80***. In the response of the T80 to amphetamine or saline, main effects of group [*F*_(2, 16)_ = 5.2; *p* = 0.02] and time [*F*_(6, 96)_ = 3.9; *p* = 0.001] were found, Figure 3. Further, an interaction effect of time and group was found [*F*_(12, 96)_ = 2.2; *p* = 0.02], Figure 3. T80 in the HRT animals increased 15 min after the amphetamine injection while in the LRT animals a significant increase was not seen until 45 min after the injection, Figure 3. Similarly, the HRT animals were significantly different from the saline controls at 25 min after injection, whereas the LRT animals were not significantly different from controls until 55 min after injection, Figure 3. However, there was no significant difference in response between the LRT and HRT animals. The saline controls did not change over time, Figure 3.

### Relationship between behavior and dopamine responsiveness

#### Reference values

The relationship between reference peaks, and the behavioral data was investigated using multivariate data analysis. A PLS with two significant components was created (*R*^2^_*X*_ = 0.53; *R*^2^_*Y*_ = 0.65; *Q*^2^ = 0.36) (Figure S6). The observed values vs. the predicted values for the reference amplitude and T80 (Figures S7A,B, respectively) indicate that the chosen behavioral parameters (Figure S6B) from the open field and MCSF test together can predict KCl-induced amplitudes and T80 values.

**Figure 4:**
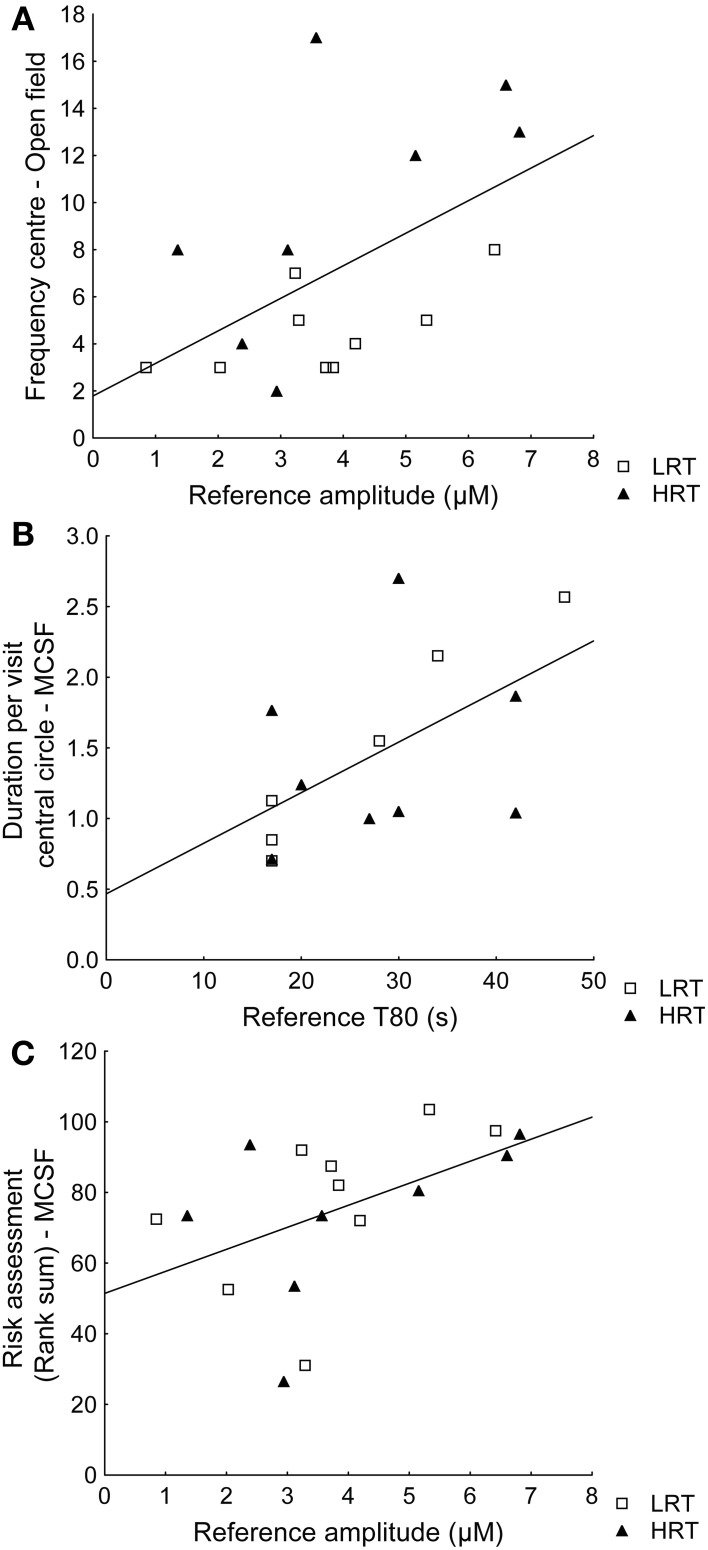
**Spearman rank order correlations in the low (LRT, *N* = 9) and high risk taking (HRT, *N* = 8) animals between (A) amplitudes and the frequency in the center of the open field test [ρ = 0.49; *p* = 0.045], (B) T80 values and the duration/frequency in the central circle of the multivariate concentric square field™ (MCSF) test [ρ = 0.60; *p* = 0.023], and (C) the risk assessment rank sum from the trend analysis of the MCSF test [ρ = 0.58; *p* = 0.016]**.

Spearman rank order correlations between behavioral parameters and amplitude or T80 values further confirmed the multivariate analysis. For example, the reference amplitude was positively correlated with the frequency in the center of the open field (ρ = 0.49; *p* = 0.045) and T80 was positively correlated with duration per visit in the central circle of the MCSF (ρ = 0.60; *p* = 0.023), Figures 4A,B respectively. However, the amplitudes were not correlated (ρ = 0.10; *p* = 0.72) to the duration (%) in the inner part of the open field. Instead risk assessment in the MCSF was positively correlated with the reference amplitudes (ρ = 0.58; *p* = 0.016), Figure 4C.

#### Amphetamine response

The relationship between the amphetamine response in T80 values and the behavioral data was also investigated using multivariate data analysis. A PLS with one significant component was created (*R*^2^_*X*_ = 0.44; *R*^2^_*Y*_ = 0.56; *Q*^2^ = 0.23) (Figure S8). The observed vs. predicted values for the response in T80 15 min after amphetamine (Figure S9) indicate that the chosen behavioral parameters from the open field and MCSF tests (Figure S8B) together can predict the amphetamine-induced increase in T80 values.

**Figure 5:**
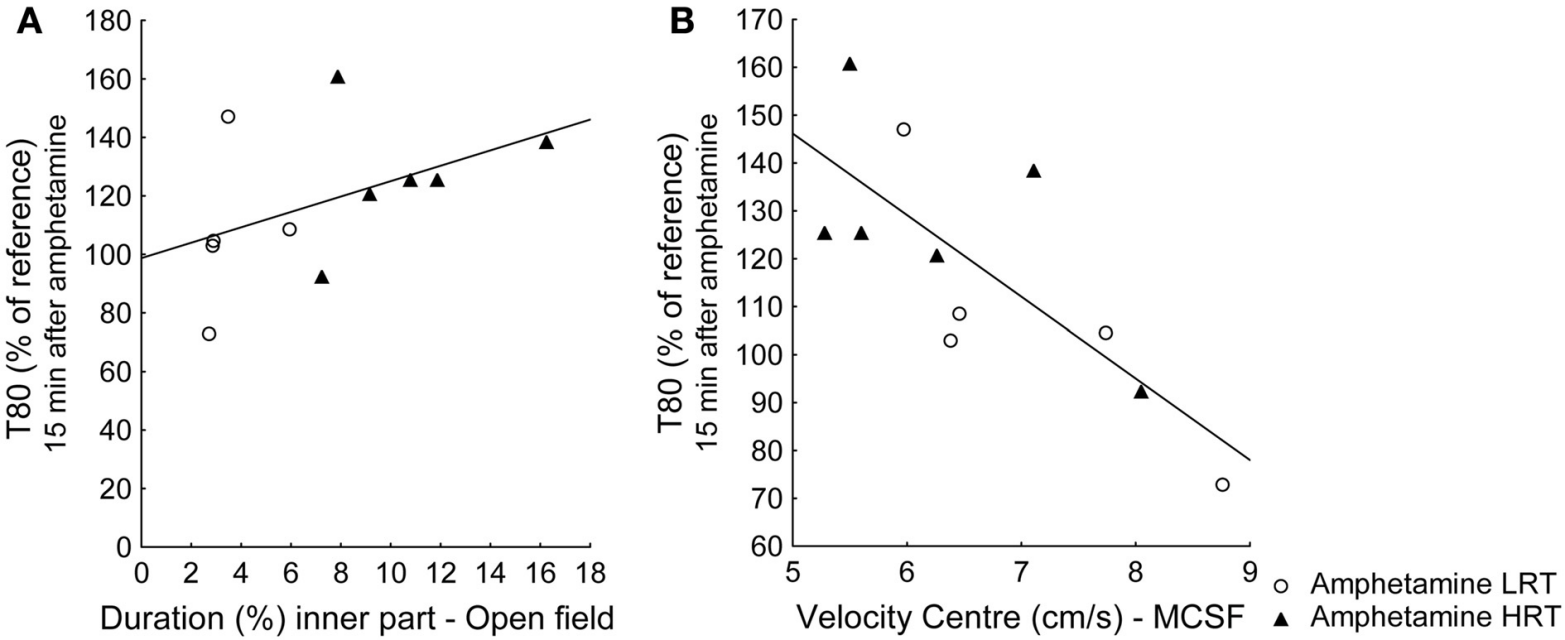
**Spearman rank order correlations of the response to amphetamine in T80 values and (A) the parameter duration (%) in the inner part of the open field, used to classify the animals into the low (LRT, *N* = 5) or high risk taking (HRT, *N* = 6) groups and (B) the velocity in the center of the multivariate concentric square field™ (MCSF) test**.

A Spearman correlation between the classifying parameter duration (%) in the inner part of the open field and the response in T80 15 min after amphetamine showed a weak, but significant, positive correlation (ρ = 0.61; *p* = 0.049), Figure 5A. The velocity in the center of the MCSF and the response in T80 15 min after amphetamine displayed a significant negative correlation (ρ = −0.75; *p* = 0.009), Figure 5B.

## Discussion

### Behavioral tests

The original classification into HR/LR animals by Piazza et al. (1989) used a circular corridor with photocells counting locomotor activity in a novel environment and the animals were then divided by a median split (Piazza et al., 1989). Others have used locomotion (Hooks et al., 1991), or rearing and sniffing in the air in an open field arena to classify the activity (Antoniou et al., 2008). By only measuring locomotor activity it may be difficult to interpret the behavior, for example whether increased activity is related to the rats' intention to explore or to search for an escape route. In the current study, the classification was based on the duration (%) in the inner part of a circular open field in order to include a risk-taking aspect (Momeni et al., 2014). For rodents, an open area includes a risk of being attacked by predators and individuals that spend more time in the inner parts of an open field can therefore be considered more risk taking. Thus, this classification is based on an active choice; to be in the center or closer to the wall. In addition the animals were tested in a multivariate setting, the MCSF, with a free choice of activities in qualitatively different zones, which enables a profiling of the animals. By classifying animals based on risk-taking and risk-assessment behavior, respectively, the different subgroups can be studied with regard to dopaminergic response. The biological function of risk assessment is to gain information about a novel situation from a cost/benefit perspective, which in the MCSF means visits to, and behaviors performed in relation to, areas associated with risk. The HRT/LRT classification correlated well with previously used classifications such as locomotion, suggesting that the HRT/LRT division can be used as an alternative in future studies of individual behavioral differences and vulnerability to drugs. Notably, the HRT/LRT classification also correlated well with the risk-taking parameters in the MCSF that were similar to the open field parameters, i.e., duration in the central circle, but not risk taking on the bridge. This shows the possibility of using the MCSF as the only test in future studies of this kind, which would eliminate the risk of carry-over effects from repeated testing. Risk-taking behavior measured by exploration of open areas, and the elevated brightly illuminated bridge is different and high risk taking in an open area is not necessarily a predictor of high risk taking on an elevated and brightly illuminated surface (Meyerson et al., 2013), in agreement with recent findings (O'leary et al., 2013). In the MCSF there are several other options to explore and this is most likely the reason for the poor predictability of open field behavior for categories other than risk taking (Meyerson et al., 2013).

### Dopamine recordings

#### Reference values

The reference amplitudes and T80 values were not different between the HRT and LRT groups, indicating that there were no differences in release capacity (Miller et al., 2012) or time course of dopamine uptake between the groups (Zahniser et al., 1998). This is in agreement with data from the dorsal striatum in a study of high and low cocaine-responding rats, which showed no differences in clearance of exogenous dopamine between the groups (Sabeti et al., 2002). The high cocaine-responding rats also displayed higher locomotor activity in response to a novel environment (Sabeti et al., 2002), consistent with the HR/LR classification (Dellu et al., 1996; Kabbaj, 2006; Blanchard et al., 2009). Whether dopamine clearance in the dorsal striatum of HR/LR rats follow the same pattern is not clear and has to our knowledge not been examined *in vivo*. A study using microdialysis showed a decreased uptake in the nucleus accumbens of HR compared to LR rats (Chefer et al., 2003). However, this was not seen in the current study, indicating regional differences in uptake between the dorsal striatum and the nucleus accumbens. Uptake has been shown to differ *in vitro* in the nucleus accumbens, but not the striatum, of LR and HR animals (Hooks et al., 1994).

It is also worth noting that chronoamperometric measurements of dopamine uptake has generally been studied by applying exogenous dopamine (Cass et al., 1993; Cass and Gerhardt, 1995), partly due to the risk of masking the dopamine transporter productivity with overwhelming concentrations of dopamine when using KCl to induce release (Miller et al., 2012) and also because amplitudes can vary in response to a set volume of KCl. However, in this study, the amplitudes of KCl-induced release were similar, allowing comparisons of the T80 values and changes in T80 could be detected, as evidenced by the response to amphetamine.

Correlations between reference amplitudes and T80 values were mainly found in parameters related to risk assessment and not to the risk-taking parameters used for classification. Thus, risk-assessment behavior was associated with innate dopamine activity. This was somewhat surprising, since there was a fairly strong correlation between the classification parameter and parameters used to classify HR/LR rats in previous studies (Dellu et al., 1996; Kabbaj, 2006; Blanchard et al., 2009) and there was some expectation that this would lead to similar findings as in HR/LR which have shown to display a good correlation between dopamine levels and the activity in a novel environment (Hooks et al., 1992). However, the current study did not measure basal extracellular levels of dopamine and it is possible that the individual variation in second-by-second release and uptake of KCl-induced overflow measured here is underlying different aspects of the behavior.

A multivariate approach was used to investigate relationships between behavior and neurochemistry. This resulted in fairly good predictions of both the reference amplitudes and the T80 values, indicating that these parameters are closely related to the behavior. The purpose for the use of multivariate data analysis was not to create a prediction model, but it is indeed an interesting prospect to be able to predict the neurochemical responses from an individual's behavior. The predictability is notable as it is known that biological parameters characterized by individual variance generate models with poorer predictability (Lundstedt et al., 1998) than for example the ones that are used in chemometrics (Wold et al., 2001).

#### Amphetamine response

When the system was challenged with amphetamine, the effect on uptake of dopamine appeared earlier in the HRT than the LRT animals. There were no significant differences between the T80 values in the two groups, but the HRT group showed a more pronounced increase in T80 compared to saline controls than did the LRT group and the increase over time was different. Correlations between amphetamine response and behavior were focused on T80 values, as there was no indication that the amplitudes changed in response to amphetamine. The PLS model of behavior and T80 response to amphetamine was not as convincing as for the reference values, and could be related to the fact that fewer animals were included in this model. It did however, point to interesting correlations between the response and behavior. For example, the T80 response at 15 min after amphetamine was positively correlated with the risk-taking classification and the velocity in center of the MCSF, which supports a more pronounced decrease in dopamine uptake after amphetamine in HRT animals. A more clear-cut difference between the groups may have been achieved if the intermediate risk takers had been removed, but this approach had demanded a larger number of animals. However, the results indicate a higher sensitivity in the HRT animals to the dopamine transport blocking effects of psychostimulants and fits with previously mentioned studies of enhanced sensitivity to locomotor effects of psychostimulants, and the readily acquired self-administration of these drugs in HR animals (Piazza et al., 1989; Hooks et al., 1991; Pierre and Vezina, 1997; Marinelli and White, 2000; Klebaur et al., 2001; Mantsch et al., 2001). Furthermore, a greater inhibition of the dopamine transporter by cocaine has been shown in rats that are high responders to cocaine (Sabeti et al., 2002).

The underlying mechanism for the increased response to amphetamine in HRT rats can only be speculated upon, but may involve differences in the number of dopamine transporters or differences in affinity of the transporter, as is indicated for the HR/LR rats (Chefer et al., 2003). The reference T80 values do not show any such differences in this study, but a lower affinity of the transporter may be compensated by a higher number of functionally active transporters in the dorsal striatum. Challenging the system with amphetamine could reveal previously masked differences, by skewing the balance between functionally active transporters and their affinity. It is also possible that the dopamine transporters in the HRT animals have a higher affinity for amphetamine, something that has not been investigated.

Differences in dopamine content in the dorsal striatum could also be a factor affecting the uptake and higher levels have been found in HR rats in this area (Antoniou et al., 2008) as well as in the nucleus accumbens (Hooks et al., 1992; Verheij et al., 2008). In this context, it is important to note the difference between tissue content, vesicular content, microdialysis data and chronoamperometric recordings. Chronoamperometry only measures the overflow of dopamine after stimulation, not the basal extracellular levels as does microdialysis, or intracellular levels outside or inside of vesicles. Amphetamine may affect all these aspects of dopamine levels (Sulzer et al., 2005), but the results from this study show no differences in amplitudes between amphetamine and saline treated animals, indicating that the dose used does not affect the dopamine overflow after potassium-induced release. Instead, our data points toward the dopamine transporter as the important factor for individual differences in the response to amphetamine in the HRT/LRT animals.

## Conclusions

By only measuring locomotor activity, as generally done in the literature, it may be difficult to interpret the behavior, for example whether increased activity is related to the rats' intention to explore or to search for an escape route. We here provide means for a more detailed behavioral analysis and show that it is possible to use a more refined behavioral test, without repeated testing, in future studies of subgroup-dependent effects. Correlations were found between the parameters used to classify the animals into HRT/LRT in this study and the parameters used to classify animals into the more widely used HR/LR classes. Risk-taking behavior in the open field was also correlated to the same type of risk taking in the MCSF. Further, the HRT rats showed a more pronounced reduction of dopamine uptake in the dorsal striatum after amphetamine compared to LRT rats. This indicates that differences in this area, which is hypothesized to be involved in the transition from drug use to addiction, may contribute to the sensitivity of these animals to the effects of psychostimulants and, in turn, proneness to addiction. Further, inherent dopamine activity was related to risk-assessment behavior, which may be of importance for decision-making and inhibitory control, key components in addiction. A broader perspective on risk-taking behavior, including aspects of risk assessment and inhibitory control and flexibility, would therefore be an interesting focus in future studies of behavioral traits linked to increased vulnerability for drug addiction.

## Author contributions

Conceived and designed the experiment and interpreted the results: Erika Roman, Ingrid Nylander, Shima Momeni, Sara Palm. Performed the experiments: Stina Lundberg, Sara Palm. Analyzed the data: Stina Lundberg, Shima Momeni, Sara Palm. Wrote the paper: Shima Momeni, Sara Palm. All authors revised and approved the final version.

### Conflict of interest statement

The authors declare that the research was conducted in the absence of any commercial or financial relationships that could be construed as a potential conflict of interest.
