# Supplementary material for: Risk-assessment and risk-taking behavior predict potassium- and amphetamine-induced dopamine response in the dorsal striatum of rats
Source: Front Behav Neurosci. 2014 Jul 15;8:236. doi: 10.3389/fnbeh.2014.00236 (PMC4097208; doi:10.3389/fnbeh.2014.00236)
Supplement: Supplementary file 1 [file DataSheet1.PDF]

Supplementary Material

Risk-assessment and risk-taking behavior predict potassium- and amphetamine-induced dopamine response in the dorsal striatum of rats

Sara Palm[*#], Shima Momeni[#], Stina Lundberg, Ingrid Nylander[+], Erika Roman[+]

Neuropharmacology, Addiction & Behaviour, Department of Pharmaceutical Biosciences, Uppsala University, Uppsala, Sweden
[#]Shared first authorship
[+]Shared senior authorship

[*]**Correspondence:** Sara Palm, Neuropharmacology, Addiction & Behaviour, Department of Pharmaceutical Biosciences, Uppsala University, P.O. Box 591, Uppsala SE-751 24, Sweden.
Sara.Palm@farmbio.uu.se

1. Supplementary Figures and Tables

1.1. Supplementary Tables

Table S1. Results from the open field test shown as median and quartile range (QR) for the low (LRT) and high risk taking (HRT) groups. Statistically significant differences, as analysed by the Mann-Whitney U-test, are shown in italics.

**Parameters**	**LRT (N=14)**		**HRT (N=15)**		**Mann-Whitney U-test**
	Median	QR	Median	QR	
F OC	11.5	7.0	18.0	6.0	*U=24.5; p<0.001*
D OC	1149	30.2	1104	35.6	*U=0.0; p<0.001*
%D OC	95.7	2.5	92.0	3.0	*U=0.0; p<0.001*
DISTANCE OC	4407	1564	4833	1569	U=76.0; p=0.21
VELOCITY OC	3.9	1.6	4.5	1.5	U=64.5; p=0.08
L IC	50.9	39.6	23.6	24.7	U=72.0; p=0.16
F IC	14.5	8.0	26.0	6.0	*U=16.5; p<0.001*
D IC	32.7	31.6	73.5	22.4	*U=11.0; p<0.001*
%D IC	2.7	2.6	6.1	1.9	*U=11.0; p<0.001*
DISTANCE IC	415	356	765	211	*U=31.0; p=0.001*
VELOCITY IC	9.9	4.9	8.9	2.6	U=81.0; p=0.31
L C	77.4	63.4	55.3	107	U=80.0; p=0.28
F C	3.0	2.0	8.0	5.0	*U=27.5; p<0.001*
D C	12.1	9.6	23.2	22.2	*U=20.5; p<0.001*
%D C	1.0	0.8	1.9	1.9	*U=20.5; p<0.001*
DISTANCE C	161	85.7	355	219	*U=22.0; p=0.03*
VELOCITY C	9.9	5.2	9.8	4.3	U=96.0; p=0.71
D IC+C	52.6	30.4	95.6	35.7	*U=0.0; p<0.001*
%D IC+C	4.4	2.5	8.0	3.0	*U=0.0; p<0.001*
TOTACT	30.0	17.0	53.0	12.0	*U=17.0; p<0.001*
TOTAL DISTANCE	4970	2091	5680	1759	*U=54.0; p=0.03*
TOTAL VELOCITY	4.2	1.8	4.8	1.5	*U=55.5; p=0.03*
REARING	27.0	28.0	45.0	20.0	*U=53.0; p=0.02*
GROOMING	3.0	3.0	3.0	2.0	U=100; p=0.84

C=center, D=duration (s), D/F=duration per frequency, F=frequency, HRT=high risk taking, IC=inner circle, L=latency (s), LRT=low risk taking, OC=outer circle, QR=quartile range, TOTACT=total activity, i.e. the sum of all frequencies.

Table S2. Results from the multivariate concentric square field™ (MCSF) test shown as median and quartile range (QR) for the low (LRT) and high risk taking (HRT) groups. Statistically significant differences, as analysed by the Mann-Whitney U-test, are shown in italics.

**Functional categories**	**Parameters**	**LRT (N=13)**		**HRT (N=14)**		**Mann-Whitney U-test**
		Median	QR	Median	QR	
General activity	TOTACT	92.0	24.0	99.5	27.0	U=79.5; p=0.59
	F TOTCORR	33.0	7.0	36.5	8.0	U=78.0; p=0.54
	F CENTER	14.0	6.0	15.5	6.0	U=56.5; p=0.10
	D CENTER	178	47.3	196	62.9	U=68.0; p=0.27
	D/F CENTER	13.3	6.1	11.8	3.7	U=71.0; p=0.34
	%D CENTER	14.9	3.9	16.3	5.2	U=68.0; p=0.27
	DISTANCE CENTER	4054	601	3941	877	U=84.0; p=0.75

	VELOCITY CENTER	6.4	1.9	6.0	1.6	U=87.5; p=0.88
	DISTANCE ARENA	9388	1754	8809	1500	U=87.0; p=0.87
	VELOCITY ARENA	7.8	1.4	7.4	1.2	U=87.5; p=0.88
	# ZONES VISITED	10.0	0.0	10.0	0.0	U=76.5; p=0.27
Exploratory activity	L LEAVE	32.9	35.8	32.2	41.9	U=75.0; p=0.45
	D TOTCORR	395	100	402	77.8	U=88.0; p=0.90
	D/F TOTCORR	11.4	3.3	11.4	4.4	U=85.0; p=0.79
	%D TOTCORR	32.9	8.4	33.5	6.5	U=88.0; p=0.90
	L HURDLE	106	123	98.4	82.0	U=87.5; p=0.88
	F HURDLE	10.0	5.0	10.0	3.0	U=81.0; p=0.64
	D HURDLE	167	47.5	166	59.4	U=90.0; p=0.98
	D/F HURDLE	19.1	3.6	16.3	7.0	U=71.0; p=0.34
	%D HURDLE	14.0	4.0	13.9	5.0	U=90.0; p=0.98
	PHOTOCELL COUNTS	4.0	15.0	4.0	7.0	U=89.5; p=0.96
	REARING	71.0	10.0	69.0	13.0	U=88.0; p=0.90
Risk assessment	L SLOPE	93.8	77.5	107	61.1	U=72.0; p=0.37
	F SLOPE	13.0	3.0	12.0	5.0	U=72.0; p=0.37
	D SLOPE	88.5	37.6	84.1	42.4	U=90.0; p=0.98
	D/F SLOPE	7.4	4.0	7.8	3.2	U=84.0; p=0.75
	% D SLOPE	7.4	3.1	7.0	3.5	U=90.0; p=0.98
	L BE	127	67.6	142	64.0	U=72.0; p=0.37
	F BE	11.0	3.0	10.0	3.0	U=75.5; p=0.46
	D BE	53.0	34.1	46.5	17.2	U=84.0; p=0.75
	D/F BE	5.4	1.9	4.9	1.9	U=91.0; p=0.98
	%D BE	4.4	2.8	3.9	1.4	U=84.0; p=0.75
	SAP TO CENTER	0.0	1.0	0.0	1.0	U=83.5; p=0.70
	OCC SAP TO CENTER	6/13		6/14		χ^2=0.03; p=0.86
Risk taking	L BRIDGE	135.9	54.7	143	67.0	U=80.0; p=0.61
	F BRIDGE	5.0	7.0	5.0	2.0	U=83.5; p=0.73
	D BRIDGE	139	48.8	145	58.1	U=74.0; p=0.42
	D/F BRIDGE	25.5	10.1	24.6	8.3	U=78.5; p=0.56
	%D BRIDGE	11.6	4.1	12.1	4.8	U=74.0; p=0.42
	L CTRCI	268	440	283	229	U=62.0; p=0.88
	F CTRCI	1.0	1.0	3.0	4.0	*U=48.5; p=0.04*
	D CTRCI	2.0	3.6	5.3	2.0	*U=47.0; p=0.03*
	D/F CTRCI	1.5	1.3	1.4	0.8	U=64.0; p=0.98
	%D CTRCI	0.2	0.3	0.4	0.2	*U=47.0; p=0.03*
	OCC CTRCI	10/13		13/14		χ^2=1.36; p=0.24
	DISTANCE CTRCI	64.5	42.7	91.3	79.6	U=39.0; p=0.11
	VELOCITY CTRCI	11.9	6.5	12.5	6.5	U=58.5; p=0.71
Shelter seeking	L DCR	210	177	179	258	U=80.0; p=0.61
	F DCR	6.0	3.0	7.0	4.0	U=69.5; p=0.30
	D DCR	150	85.0	137	114	U=88.0; p=0.90
	D/F DCR	21.5	5.6	20.4	8.5	U=77.0; p=0.51
	%D DCR	12.5	7.1	11.4	9.5	U=88.0; p=0.90
Anxiety-like behaviour	F RISK/SHELTER INDEX[1]	-0.11	0.10	0.13	0.38	U=81.0; p=0.64
	D RISK/SHELTER INDEX[2]	0.09	0.32	0.04	0.49	U=83.0; p=0.72
Impulsive-like behaviour	SLOPE/BRIDGE INTERVAL[3]	-0.15	0.68	-0.14	0.61	U=90.0; p=0.98
Other	GROOMING	1.0	2.0	1.0	1.0	U=79.5; p=0.58

OCC GROOMING	8/13		11/14		χ^2=0.94; p=0.33
OCC BOLI	0/13		0/14		
URINE	1.0	2.0	1.0	2.0	U=77.0; p=0.50
OCC URINE	10/13		10/14		χ^2=0.11; p=0.74

[1]F RISK/SHELTER INDEX = (F BRIDGE – F DCR)/(F BRIDGE + F DCR)
[2]D RISK/SHELTER INDEX = (D BRIDGE – D DCR)/ D BRIDGE + D DCR)
[3]SLOPE/BRIDGE INTERVAL = (L SLOPE – L BRIDGE)/L SLOPE
BE=bridge entrance, CTRCI=central circle, DCR=dark corner room, D=duration (s), D/F=duration per frequency (s), F=frequency, L=latency (s), OCC=occurrence, SAP=stretched attend posture, TOTACT=total activity, i.e. the sum of all frequencies, TOTARENA=total arena, TOTCORR=total corridor, i.e. the sum of all corridors.

Table S3. Reference amplitudes (μM) and T80 (s) before subcutaneous injections of saline or amphetamine in low risk taking (LRT) and high risk taking (HRT) animals. All values are expressed in mean ± SEM.

	**Saline (N=7)**			**Amphetamine LRT (N=6)**			**Amphetamine HRT (N=7)**		
Amplitude 1 (μM)	3.9	±	0.8	3.5	±	0.9	4.4	±	0.7
Amplitude 2 (μM)	4.1	±	0.9	3.6	±	0.9	4.2	±	0.7
Amplitude 3 (μM)	4.0	±	0.8	3.5	±	0.9	4.2	±	0.8
T80 1 (s)	29.6	±	5.2	30.2	±	5.7	22.9	±	2.0
T80 2 (s)	28.0	±	4.1	27.3	±	4.6	24.0	±	2.2
T80 3 (s)	26.3	±	3.6	25.7	±	5.8	23.9	±	2.5

Table S4. Response over time (%) after subcutaneous injections of saline or amphetamine in low risk taking (LRT) and high risk taking (HRT) animals. Response amplitudes are normalised to per cent (%) of reference amplitudes. All values are expressed in mean ± SEM.

	**Saline (N=7)**			**Amphetamine LRT (N=6)**			**Amphetamine HRT (N=7)**		
5 min (%)	93	±	5.4	95	±	3.7	92	±	2.5
15 min (%)	95	±	3.7	103	±	8.3	95	±	5.1
25 min (%)	86	±	7.2	104	±	8.0	103	±	6.9°
35 min (%)	89	±	6.3	106	±	9.3	98	±	5.9
45 min (%)	86	±	6.7	104	±	11.9	98	±	7.8
55 min (%)	79	±	10.8°	95	±	8.2	92	±	8.4&
65 min (%)	75	±	11.3°°#	95	±	11.8	86	±	7.8§

° $p < 0.05$, °° $p < 0.01$ compared to the 5-min time point, # $p < 0.05$ compared to time points 15, 25, 35 and 45, & $p < 0.05$ compared to the 25-min time point, § $p < 0.05$ compared to time points 25, 35 and 45 (repeated measures ANOVA followed by Fisher's LSD test).

1.2. Supplementary Figures

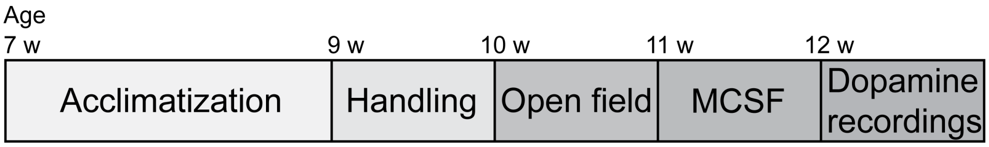

Figure S1. An overview of the sequence of handling, tests and the ages of the rats at the respective test. MCSF = multivariate concentric square field™ test, w = weeks.

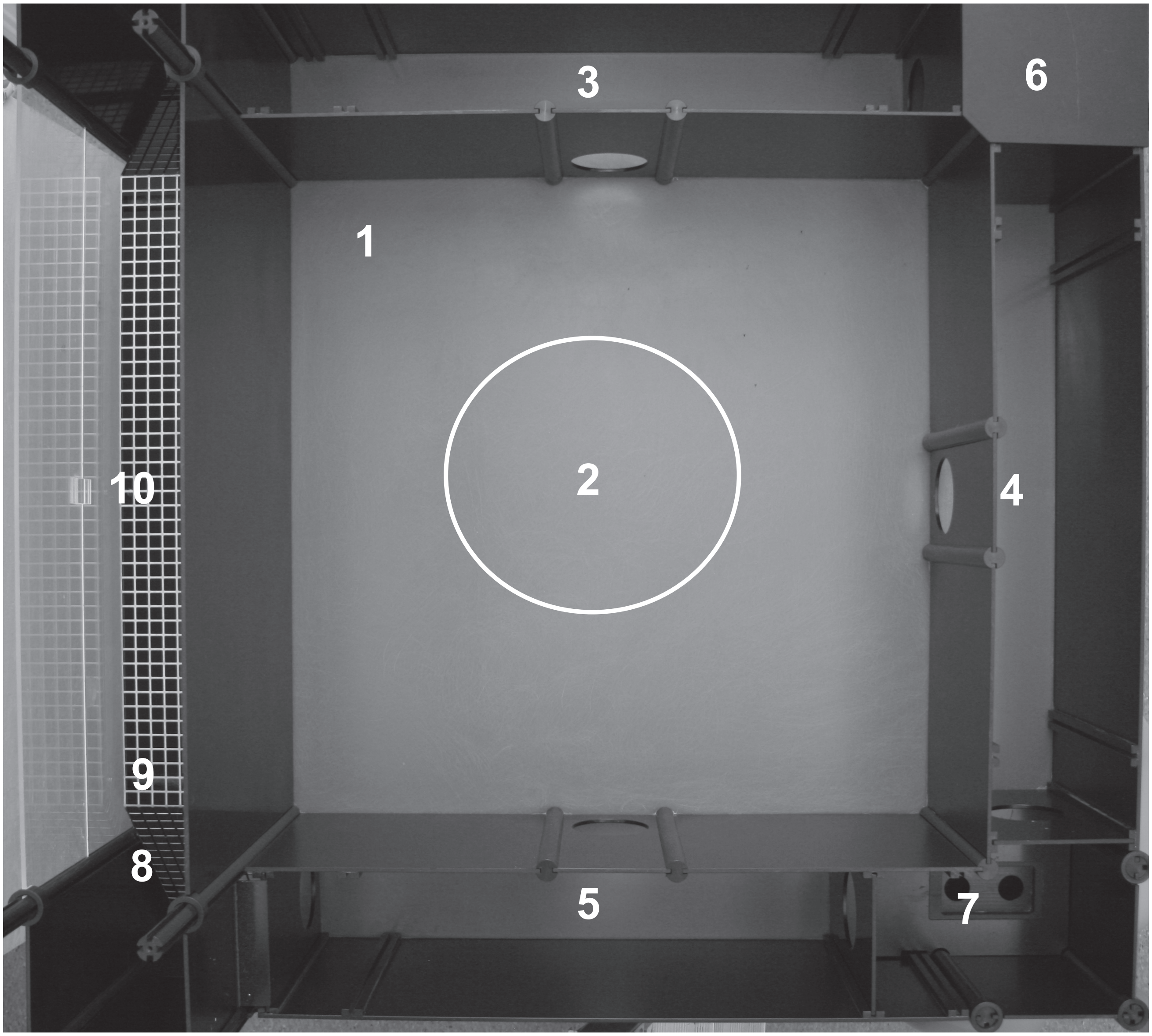

Figure S2. The multivariate concentric square field™ test arena (100 × 100 cm) and the defined zones, numbered as follows: 1. Center, 70 × 70 cm, open area; 2. Central circle, 25 cm diameter, risk area; 3-5. Corridors, transit areas; 6. Dark corner room (DCR), area for shelter seeking; 7. Hurdle, high passage to hole board with photocell to count head dips, exploratory incentive; 8. Slope, leading up to Bridge, risk assessment area; 9. Bridge entrance, risk assessment area; 10. Bridge, elevated and illuminated, risk area.

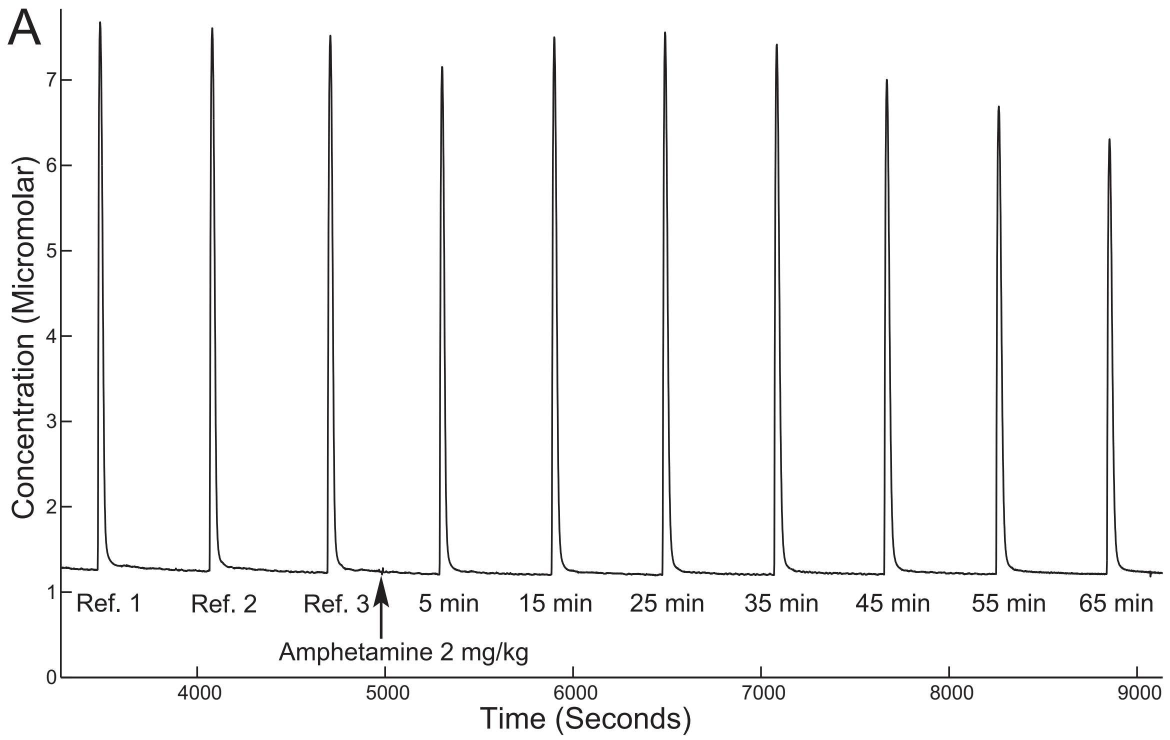

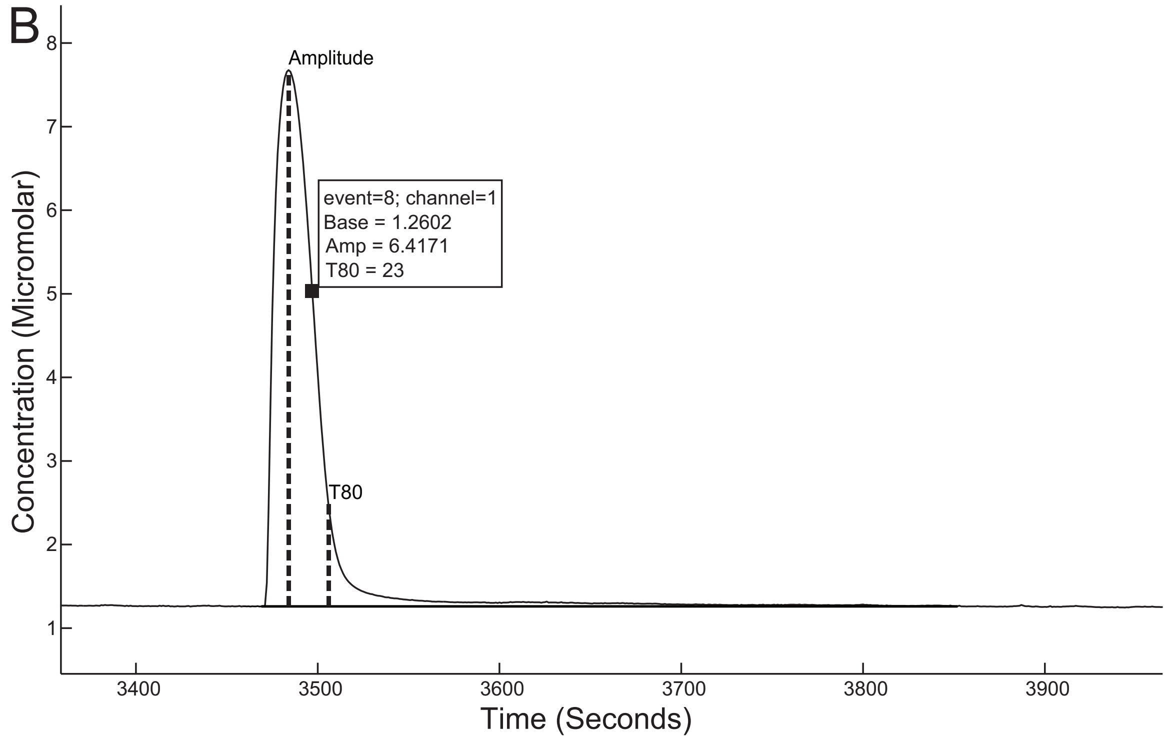

Figure S3. A) A representative trace of the oxidation current for a rat receiving amphetamine and B) a close-up of the first reference peak for the same animal showing how amplitude and T80 were calculated. Amp = amplitude, Base = baseline, Ref = reference.

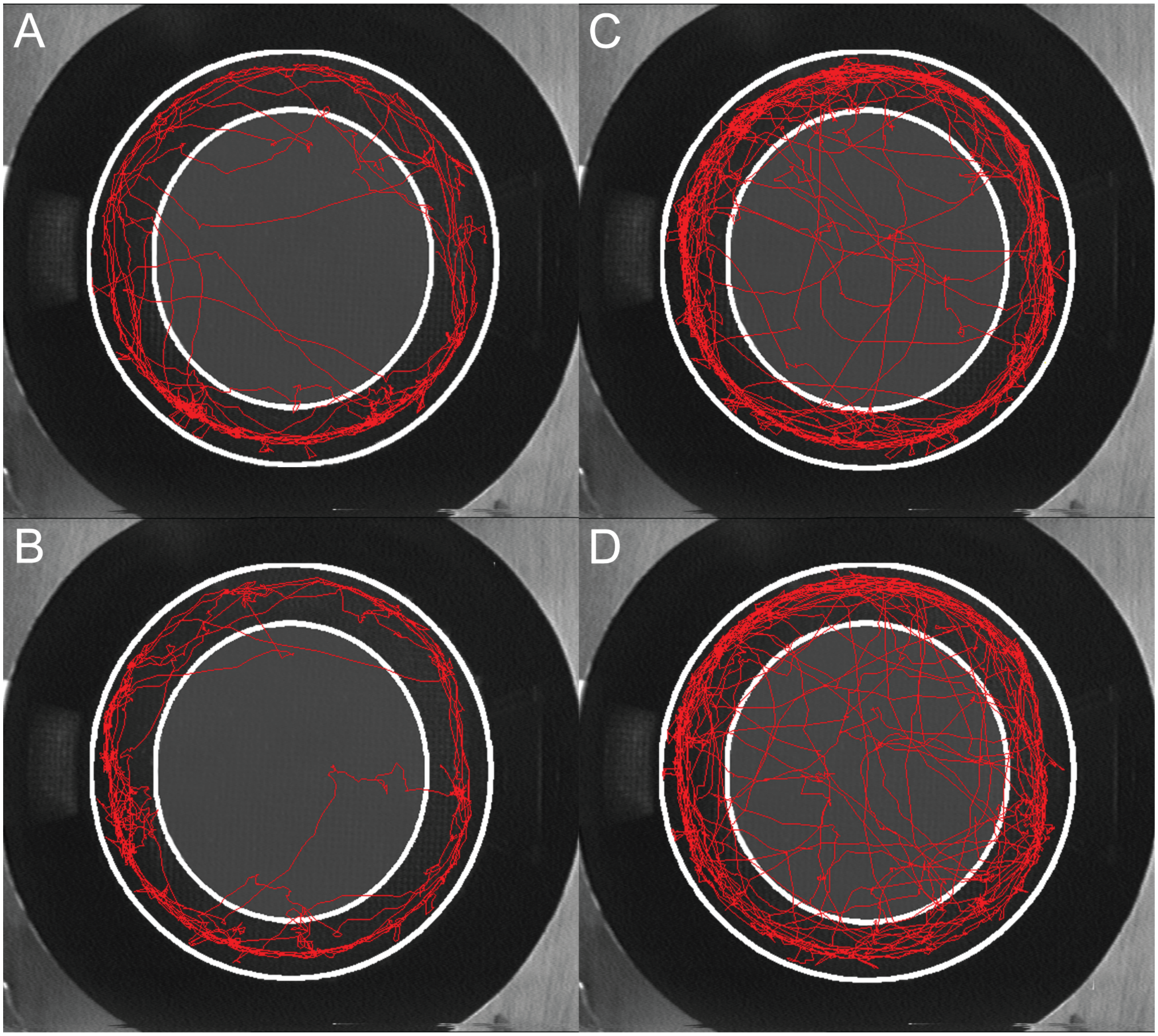

Figure S4. Traces (in red) of open field recordings for A) the median in the low risk taking group, B) the least risk taking rat, C) the median in the high risk taking group and D) the most risk taking rat. The outer white line shows the entire arena (90 cm in diameter) and the inner white line shows the center (30 cm in diameter) of the arena.

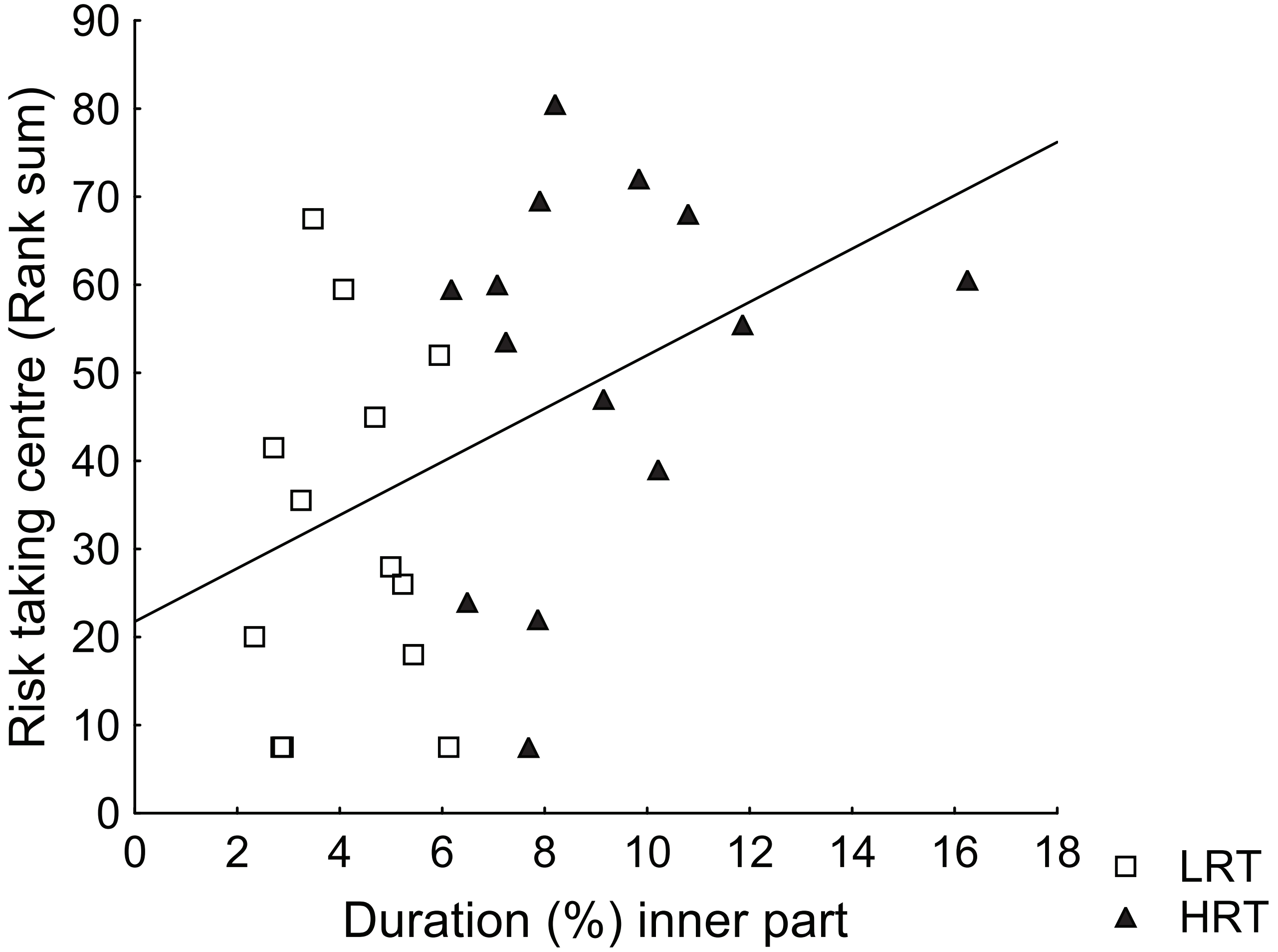

Figure S5. A Spearman rank order correlation between the central circle-related risk taking in the multivariate concentric square field™ (MCSF) test and duration (%) in the inner part of the open field, used to classify the animals into the low (LRT, N=13) or high risk taking (HRT, N=14) groups [ρ=0.49; p=0.010].

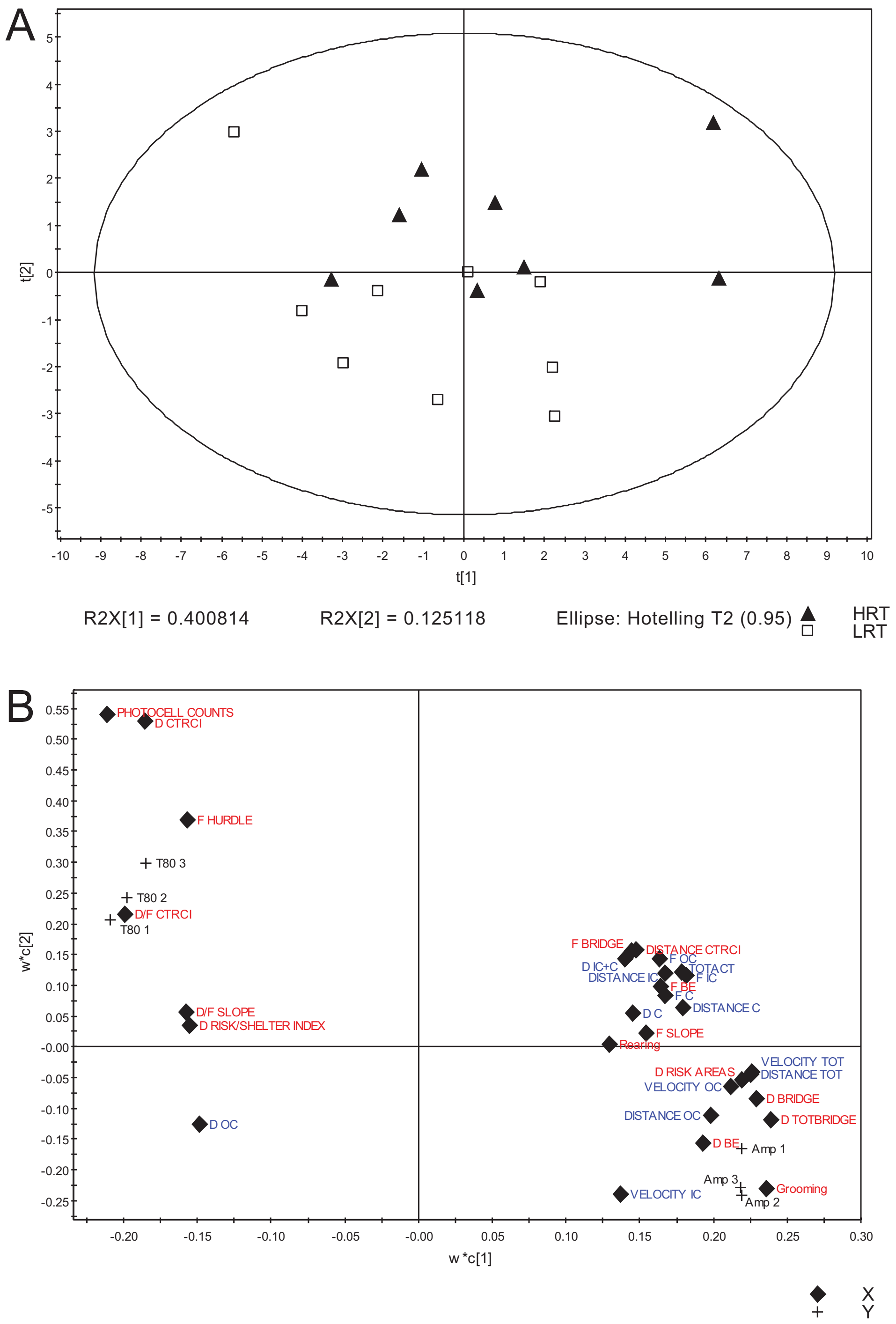

Figure S6. The **(A)** score plot and **(B)** loading plot for the partial least squares projections to latent structures (PLS) for chosen behavioral parameters, and reference amplitudes and T80 values [R^2_X=0.53; R^2_Y=0.65; Q^2=0.36]. Blue denotes parameters from the open field and red denotes parameters from the multivariate concentric square field™ (MCSF) test. C=center, CTRCI=central circle, DCR=dark corner room, D=duration (s), D/F=duration per frequency (s), F=frequency, HRT=high risk taking (N=8), IC=inner circle, L=latency (s), LRT=low risk taking (N=9), OC=outer circle, TOTACT=total activity, i.e. the sum of all frequencies, TOTCORR=total corridor, i.e. the sum of all corridors.

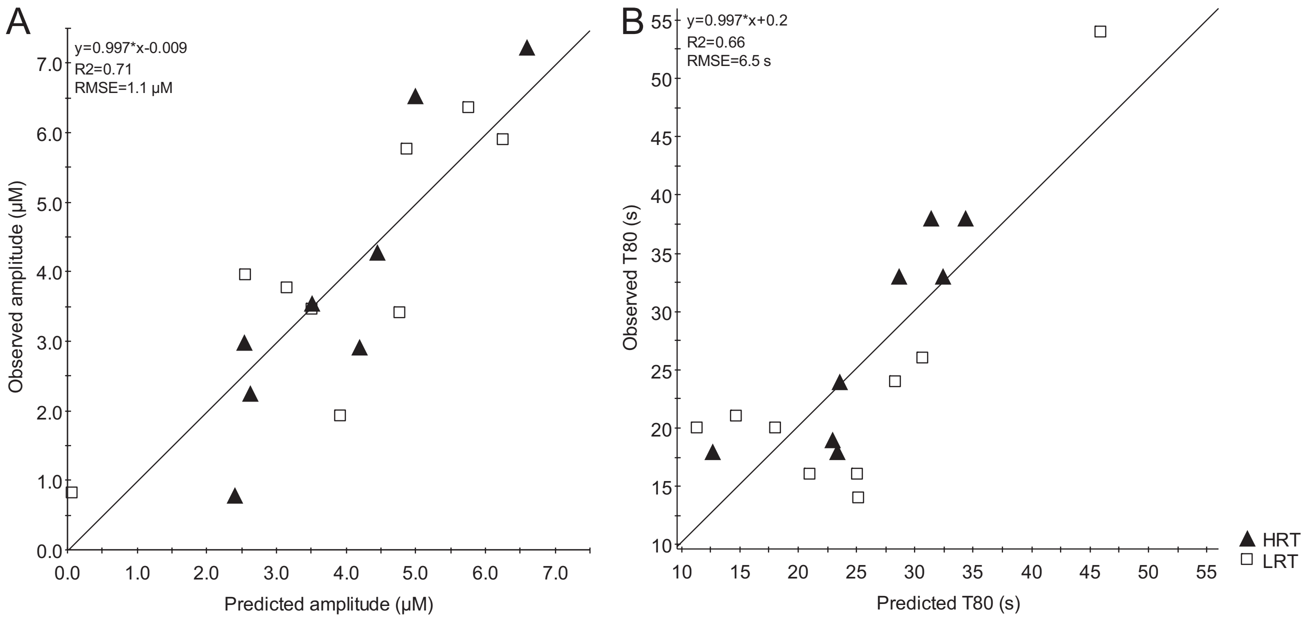

Figure S7. Observed versus predicted **(A)** reference amplitudes and **(B)** reference T80 values from the partial least squares projection to latent structures (PLS) based on parameters from the open field and multivariate concentric square field™ (MCSF) tests (Figure S4B) and the chronoamperometric dopamine recordings in low (LRT, N=9) and high risk taking (HRT, N=9) animals. The fit of the respective regression line and the root mean square error (RMSE) is shown in the upper left corner of each graph.

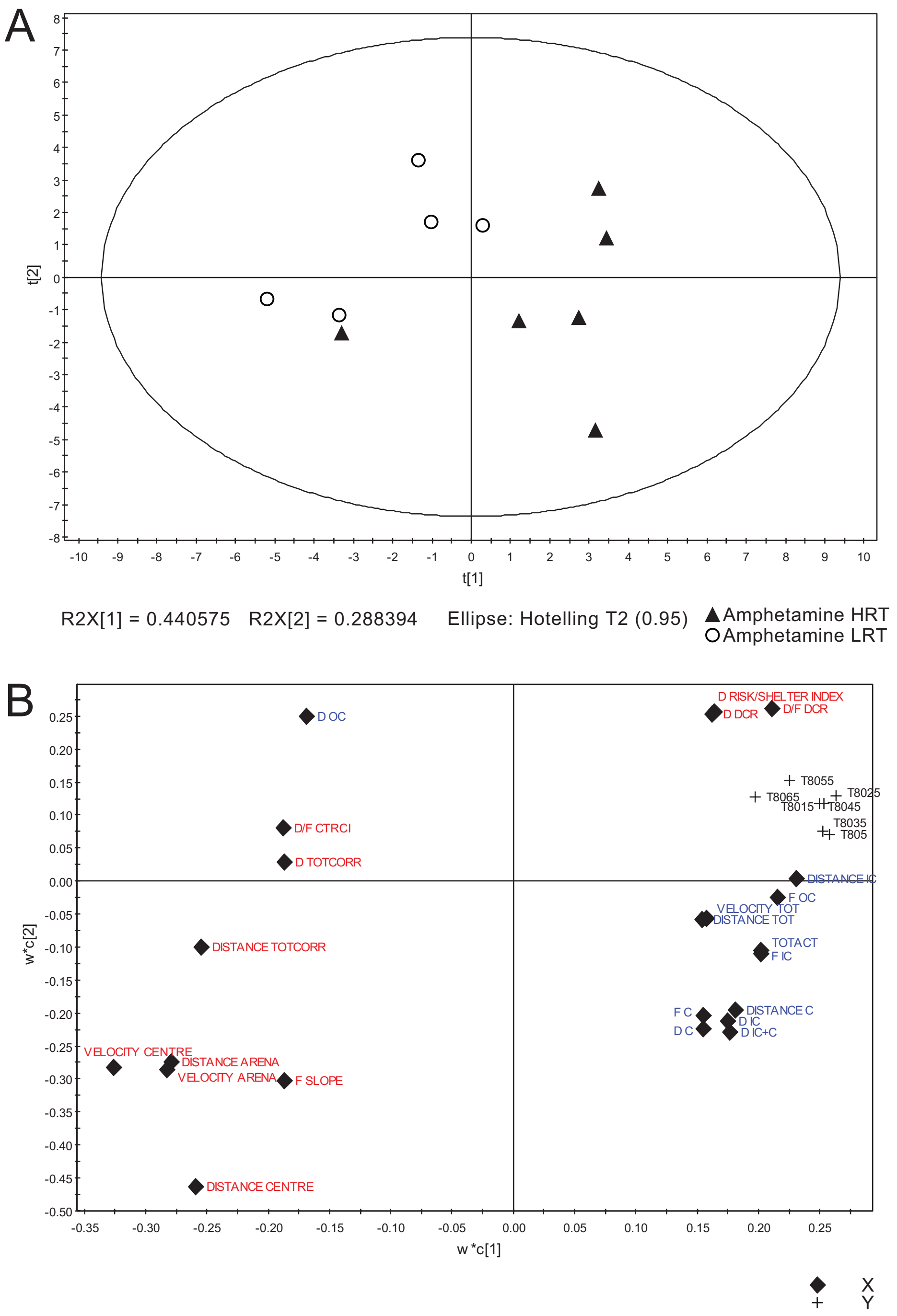

Figure S8. The **(A)** score plot and **(B)** loading plot for the partial least squares projections to latent structures (PLS) for chosen behavioral parameters and T80 response to amphetamine [R^2_X=0.44; R^2_Y=0.56; Q^2=0.23]. Only the first component was significant. Blue denotes parameters from the open field and red denotes parameters from the multivariate concentric square field™ (MCSF) test. C=center, CTRCI=central circle, DCR=dark corner room, D=duration (s), D/F=duration per frequency (s), F=frequency, HRT=high risk taking (N=6), IC=inner circle, L=latency (s), LRT=low risk taking (N=5), OC=outer circle, TOTACT=total activity, i.e. the sum of all frequencies, TOTCORR=total corridor, i.e. the sum of all corridors.

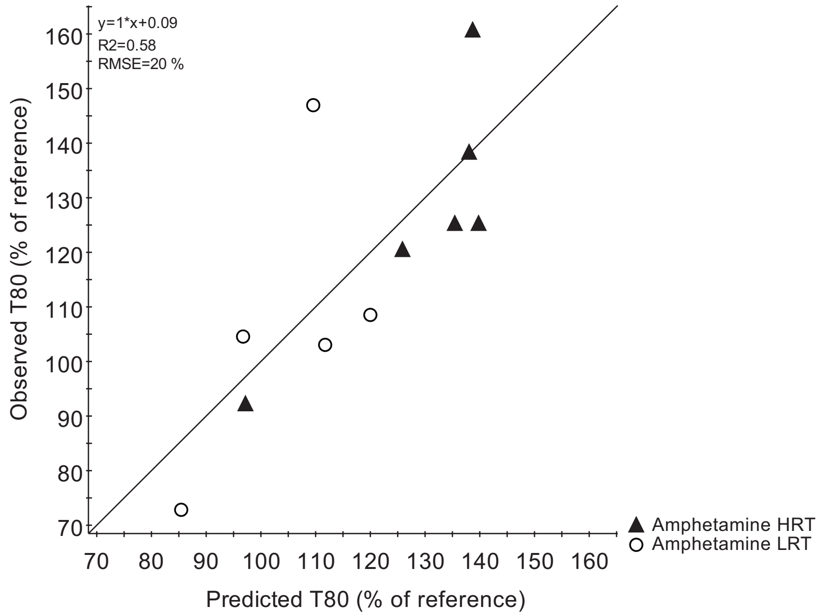

Figure S9. Observed versus predicted response to amphetamine in T80 values from the partial least squares projection to latent structures (PLS) based on parameters from the open field and multivariate concentric square field™ (MCSF) tests (Figure S6B) and the chronoamperometric dopamine recordings in low (LRT, N=5) and high risk taking (HRT, N=6) animals. The fit of the regression line and the root mean square error (RMSE) is shown in the upper left corner.
